# When ethnographic work turns into distant screen visits: A note on flexible inflexibility during the COVID-19 pandemic

**DOI:** 10.1177/14639491221107361

**Published:** 2022-06-12

**Authors:** Helena Sandberg, Ebba Sundin, Ulrika Sjöberg

**Affiliations:** 5193 Lund University, Sweden; 3694 Halmstad University, Sweden; 5264 Malmo University, Sweden

**Keywords:** Children, digital media practices, digital screen visits, ethnographic fieldwork, ethnographic methodologies

## Abstract

This colloquium shares experiences from doing ethnographic fieldwork with young children and the challenges that followed due to the COVID-19 pandemic. The project DIGIKIDS Sweden has its focus on very young children (birth to three years) and their engagement with digital media technologies in their homes. The pandemic put the project on hold, but in the families where the fieldwork had already started, the authors decided to change the methods of data collection. Digital screen visits were introduced and, at first, this seemed to be flexible, and they adjusted to the new environment. At the same time, this flexibility also became an inflexible experience due to the use of technology.

## Introduction

In the early spring of 2020, we were in the middle of collecting data when the COVID-19 pandemic put our work on hold. In this colloquium, we would like to share some of our experiences from doing ethnographic fieldwork with young children and their families, and the challenges that followed due to the pandemic.

In the DIGIKIDS Sweden project, we investigate very young (birth to three years) children's appropriation of (Silverstone et al., 1992) and engagement with digital media technologies in their home settings. This is an underestimated research topic within media and communication studies, despite the fact that nowadays screen activity is a natural part of modern childhood and parenthood. Children are familiar with digital media technologies in their homes from an early age, and empirical research with infants and toddlers is emerging (Kumpulainen and Gillen, 2017).

The research design of qualitative work is, by nature, flexible. We use the term ‘ethnographic work’ in the sense of describing methods, and we agree with Wolcott’s (2008) urge for researchers to separate ‘between doing ethnography and borrowing ethnographic techniques’ (44). This does not mean that ethnography has a clear-cut definition; the field has developed in several directions, and Wolcott acknowledges that ethnography ‘has long since slipped out from under the anthropological tent’ (45).

The pandemic changed the conditions for ‘traditional’ fieldwork, and there is a lot to learn from how researchers have adapted to the changing circumstances. The pandemic has, in fact, pushed the use of new fieldwork methodologies. Researchers working in the field needed to adapt to the new circumstances and be prepared to be flexible and agile, while also responsible and ethically reflective (Sandberg and Gillen, 2021). The pandemic put us in new, unforeseen situations that were difficult to circumvent.

In our research, we apply the ethnographic perspective of ‘A day in the life’ approach (Gillen et al., 2007), which consists of three visits to the home of each child. It includes interviews with the families, observations and videoing of the child in focus during a full day, surveys (mapping media access), drawings of the domestic space, and a follow-up interview based on co-viewing a video compilation of the recorded day. We work in pairs with the ambition to cover as much as possible of the multiple aspects in children's everyday life. Included in our responsibilities is also to ensure that the technology works and, finally, but not least, that the child in focus gets our full attention. The design also has a lot in common with what has been labelled ‘mobile ethnography’ (Novoa, 2015). Our aim has been similar: to follow the child and their interactions with family members and digital media over a day, and to try to experience their everyday life with all its means of being present.

## Socially distanced fieldwork during the pandemic

At the beginning of 2020, we had visited six families. We still had the third visit to make to five of the families. We were scheduled to do the videoing of one of the families just a few days after the pandemic was declared.

We needed to protect our research participants and ourselves, and to follow the rules of social distancing. We decided to share the videos on memory sticks, which were sent to the families by ordinary mail, instead of co-viewing the video compilations with the families in their homes. The planned home visits, scheduled for at least two hours including the family conversation/interview, were promptly transformed into distant screen visits – a conversation over Zoom. It turned out that the families responded positively to the Zoom interactions, which was more than we expected. At this time, the Zoom application was quite new to many of us, and we were not used to this kind of screen interaction in the format of family interviews. In many ways, the Zoom application offered appealing opportunities for social interaction. ‘Zoom fatigue’ had not yet been heard of.

The conversations probed the children's reactions and the parents’ reflections on the pre-viewed video. The first distant screen visit was scheduled for two hours, as originally agreed. In some families, we had a break when needed; for others, we proceeded with a continuous interaction, as we would have done in a face-to-face meeting. With some families, we encouraged the older participating children to join in the conversation. For ethical and security reasons, we did not record our visits and interviews via Zoom. An external recorder for the audio was used, and the interviews were transcribed afterwards.

Doing the home visits and follow-up interviews using Zoom appeared at first to be an appealing and good decision, so that we would not risk losing data or having families drop out due to the pandemic. We were all eager to follow through and finalise the data collection, and the families seemed happy with the solution offered. However, it turned out that carrying out family conversations/interviews and home visits through a digital screen was not as easy as it had first appeared. In retrospect, we realise that we learned a lot, but we would do it differently if we decided to pursue this approach again. In this colloquium, we address what made this research process complicated.

## Complications of digital screen visits

When we visit homes, the focus is on the children, since our interest is to address their voice and agency. When engaged in a digital screen visit, everything became media-centric and focused on the technology at hand: the tiny screen and the laptop or the platform used by the families. The children and adults struggled to be seen, heard and paid attention to, which made it rather difficult to interact in front of the screen. The very young children wanted to touch the screen and the keyboard, and one even accidently closed the session. We were able to restart the meeting but felt it had lost momentum. It was also difficult to meet the needs and interests of the children in focus, as it became difficult to keep their attention due to simultaneous interactions with the rest of the family and sometimes quarrels with younger siblings who were present at the interview.

It was also more difficult to relate to the children in focus. It is harder to talk and give extra attention on a screen. We had sent a special invitation letter to the children in focus with a simple explanation and some pictures of the digital screen, as well as of ourselves, to remind them about the upcoming digital meeting. Still, it was difficult to connect, since we did not know how much the children remembered us from the previous visits, and we found it difficult to get their attention and responses. It also became obvious that the children in focus were easily distracted and were paying little attention to our questions. We asked a three-year-old girl sitting in front of the screen together with her parents about watching the video compilation, but got no response from her. She was silent and not very interested in us. Then, suddenly, she burst out in a loud voice that there had been a fire at her nursery school. One of us researchers asked if this was one (referring to the nursery school) where she was now. She became silent and then made a face on the screen and answered that she was here (in front of the screen) not at the nursery school.

There were things that could have been handled more smoothly if we had been present in the room. The body language and atmosphere (e.g. potential tension in the air between the participants) were difficult to detect or complex to perceive. The handling of urgent situations or accidents, or attending to children's different needs, also became very difficult being in front of a screen (e.g. in one family, the older sibling pushed the baby brother, there were screams and the baby accidently fell onto a table). The screen was blurring the distance factor but preventing closeness and presence in the room. It made communication difficult for our participants as well as for us as researchers. The families and children were crouched in front of one camera and screen, taking turns, struggling to make it work, responding and reflecting aloud while the parents were always keeping an eye on the children moving around the screen or eager to tap on the technology. We were at a COVID-safe distance from each other, sitting in different places behind different screens, but on Zoom we appeared to be quite close, and, as a team, we still did not have the full capacity to support each other or the families we wanted to be close to and engaged with. This caused frustration and dissatisfaction.

The parents used different strategies to handle the situation during these digital visits and worked hard to keep their children close to the screen – probably more so than if we had been in the room with them. The parents encouraged their children to engage by simplifying and translating the questions, and prompting them to elaborate on their responses. They also enticed them with snacks and ice cream, and other things, such as being allowed to watch a video in a nearby room or play with a tablet that was off limits at other times.

It was not all bad, but it was definitely challenging. We did see opportunities for engaging with young children via digital screens. The technology offers direct and unexpected opportunities to learn more about children's digital literacy and interest in online interactions. Another advantage is the possibility to take short breaks when needed. During a physical visit, if a situation calls for letting children have a moment alone with their parent(s), it takes up time to leave the home for a short walk in the neighbourhood or to wait outside until it is appropriate to continue the conversation.

The kind of fieldwork we aim for cannot be seamlessly replaced by digital substitutes. In fact, with the digital screen technology – in this case, Zoom – we as researchers potentially introduced new software technology to young children and made them interact in ways not experienced before.

## Flexible inflexibility

The pandemic forced us to be methodologically creative and adapt to the novel situation that we found ourselves in, which is a common approach in qualitative fieldwork. Thus, we introduced digital screen visits to be flexible and adjust. Simultaneously, this flexibility became inflexible as a consequence of the technology. The screen limited our interactions and data collection in various ways, as described above. At the same time, we were challenged by inflexible flexibility since we were limited by formal ethics procedures, which constrained our creativity in using certain methods. For example, if a research project needs ethical clearance and has been approved, researchers are not permitted to go beyond what has been described and accepted in the ethical approval. The formal ethics process may actually prevent a qualitative researcher from being innovative and trying out new things and approaches in any project without an amendment application to the Swedish ethical review authority. This process frequently takes time and delays data collection. While the purpose of this is for the benefit of the research and the participants, it nevertheless risks hindering creativity and methodological developments.

In our case, we decided to put any further data collection on hold, and between June 2020 and November 2021 we paused the fieldwork. We were able to continue the project in December 2021, but with an awareness of changing restrictions and recommendations. Also, we have become more aware of having to reschedule at short notice due to COVID-19 symptoms and illness. As researchers during a pandemic, we need to constantly plan and prepare for the unforeseen. Never has the need to be flexible while also accepting the inflexible been so important.

When the data collection is complete by the end of 2022, we will have not only a variety of data on families with young children and their engagement with digital media technologies, but also different types of data sets to be compared from the perspective of the digital media technologies themselves. Similarities or differences may be found when comparing the data sets, and also when comparing them from a pre- and post-COVID perspective. 
